# Smed454 dataset: unravelling the transcriptome of *Schmidtea mediterranea*

**DOI:** 10.1186/1471-2164-11-731

**Published:** 2010-12-31

**Authors:** Josep F Abril, Francesc Cebrià, Gustavo Rodríguez-Esteban, Thomas Horn, Susanna Fraguas, Beatriz Calvo, Kerstin Bartscherer, Emili Saló

**Affiliations:** 1 Departament de Genètica, Facultat de Biología, Universitat de Barcelona (UB), Av. Diagonal 645, edifici annex, planta 1, 08028, Barcelona, Catalunya, Spain; 2 Institut de Biomedicina de la Universitat de Barcelona (IBUB), Av. Diagonal 645, edifici annex, planta 1, 08028, Barcelona, Catalunya, Spain; 3 Division of Signaling and Functional Genomics German Cancer Research Center (DKFZ), Im Neuenheimer Feld 580, 69120 Heidelberg, Germany; 4 Max Planck Research Group Stem Cells and Regeneration Max-Planck-Institute for Molecular Biomedicine, Von-Esmarch-Strasse 54, 48149 Muenster, Germany

## Abstract

**Background:**

Freshwater planarians are an attractive model for regeneration and stem cell research and have become a promising tool in the field of regenerative medicine. With the availability of a sequenced planarian genome, the recent application of modern genetic and high-throughput tools has resulted in revitalized interest in these animals, long known for their amazing regenerative capabilities, which enable them to regrow even a new head after decapitation. However, a detailed description of the planarian transcriptome is essential for future investigation into regenerative processes using planarians as a model system.

**Results:**

In order to complement and improve existing gene annotations, we used a 454 pyrosequencing approach to analyze the transcriptome of the planarian species *Schmidtea mediterranea *Altogether, 598,435 454-sequencing reads, with an average length of 327 bp, were assembled together with the ~10,000 sequences of the *S. mediterranea *UniGene set using different similarity cutoffs. The assembly was then mapped onto the current genome data. Remarkably, our Smed454 dataset contains more than 3 million novel transcribed nucleotides sequenced for the first time. A descriptive analysis of planarian splice sites was conducted on those Smed454 contigs that mapped univocally to the current genome assembly. Sequence analysis allowed us to identify genes encoding putative proteins with defined structural properties, such as transmembrane domains. Moreover, we annotated the Smed454 dataset using Gene Ontology, and identified putative homologues of several gene families that may play a key role during regeneration, such as neurotransmitter and hormone receptors, homeobox-containing genes, and genes related to eye function.

**Conclusions:**

We report the first planarian transcript dataset, Smed454, as an open resource tool that can be accessed via a web interface. Smed454 contains significant novel sequence information about most expressed genes of *S. mediterranea*. Analysis of the annotated data promises to contribute to identification of gene families poorly characterized at a functional level. The Smed454 transcriptome data will assist in the molecular characterization of *S. mediterranea *as a model organism, which will be useful to a broad scientific community.

## Background

One of the challenges that medical research must address in the near future is to understand why some animals are able to regenerate complex structures, including eyes and even whole bodies, from small body fragments, while others are not. With the recent emergence of the field of regenerative medicine, the future biomedical ramifications of the study of animal regeneration are obvious.

Freshwater planarians are a classic model for studying the fascinating process of regeneration [1-4] because they are capable of re-building a complete organism from almost any small body fragment. This is made possible by a unique population of adult somatic stem cells called neoblasts. During regeneration and constant homeostatic cell turnover, neoblasts differentiate into all cell types, including germ cells in sexual species [5,6]. In recent years, several studies have begun to unravel the mechanisms by which regeneration is regulated at the molecular level. For example, different genes have been shown to play pivotal roles in axon guidance and neurogenesis [7], the regulation of neoblast proliferation and differentiation [8,9], and the re-establishment and maintenance of the anteroposterior (AP) and dorsoventral (DV) body axes [10]. *Schmidtea mediterranea *and *Dugesia japonica *are the two planarian species most often used in regeneration studies. There are about 78,000 ESTs (Expressed Sequence Tags) for *S. mediterranea *in NCBI generated in different projects [11,12]. Those sequences were clustered to produce a set of 10,000 putative mRNAs which are available from the NCBI Unigene database [13]. The *S. mediterranea *genome has also been sequenced and assembled [14] at the Genome Sequencing Center at Washington University in St. Louis (WUSL, USA) after approval of a white paper [15]. However, because of this genome's internal complexity (67% A+T, [16]) and the lack of a BAC library, its completeness and assembly still needs improvement. A step towards this end was taken when the *S. mediterranea *genome and EST information were integrated and approximately 30,000 genes were predicted using an annotation pipeline called MAKER[16]. Those gene models, together with ~9,000 mRNAs generated using next-generation sequencing technology, were mapped on the planarian genome and used to improve the assembly [17]. The current assembly contains 43,673 contigs. These are accessible, together with the MAKER annotation data, in the *S. mediterranea *genome database (SmedGD; [17]).

In order to expand our knowledge of the planarian transcriptome and to provide a new tool that can be used to improve the *S. mediterranea *genome annotation, we generated a new transcriptome dataset using 454 pyrosequencing technology [18]. The Smed454 dataset can be freely accessed via a website, and the complete sequence data can be downloaded by anyone from there. Mapping of the Smed454 ESTs onto the genome scaffolds shows that the Smed454 dataset contains more than 3 million nucleotides sequenced *de novo*. In addition, this mapping extends and connects currently fragmented genomic contigs. Finally, GO annotation of the Smed454 dataset assigns candidate functions to those sequences and facilitates their grouping into distinct gene families. In this way, whole gene families can be analyzed for putative roles in planarian regeneration. Thus we are confident that the Smed454 dataset will improve our understanding of how planarian regeneration works at the molecular level.

## Results and Discussion

### Construction and sequencing of the Smed454 dataset

In order to obtain the most representative set of planarian genes expressed under different physiological conditions, total RNA was isolated from a mixture of non-irradiated and irradiated intact and regenerating planarians (see Methods). We used planarians regenerating both head and tail to identify the genes specifically expressed in a tissue-specific manner. Similarly, planarians at different stages of regeneration were used in order to isolate genes with different temporal expression profiles. Irradiation destroys planarian neoblasts within 1-2 days, and the animals die within a few weeks because they cannot sustain normal cell turnover. By including irradiated animals, potential transcripts specifically expressed under those conditions will be contained in the 454 dataset.

**Figure 1:**
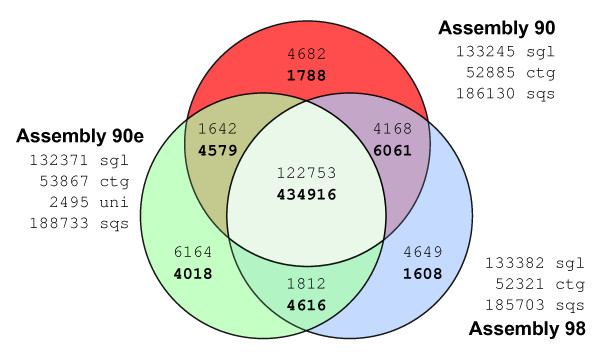
**Overlap-analysis of Smed454 assemblies**. Comparison of the 454-sequencing reads taken into account to build each Smed454 dataset. Venn-diagram numbers in plain format correspond to singleton reads, while numbers in bold correspond to sequencing reads that were assembled into Contigs. About 4,000 raw reads where split into two or more fragments, due to quality clipping. However, only distinct raw read identifiers, after removing the fragment suffix, were used to produce this figure. sgl: total number of singletons for that assembly; ctg: total number of contigs; uni: number of NCBI Unigene sequences not assembled into a contig (90e only); sqs: total number of sequences for a given dataset.

Using 454 pyrosequencing, 601,439 sequencing reads with an average length of 327 bp were obtained. After sequence cleaning to remove vector contamination, the remaining 598,435 sequences were assembled using different cut-off values for sequence similarity (90%, 95% and 98%). In addition, our 454 sequence reads were assembled together with the ~10,000 *S. mediterranea *UniGene set available at NCBI, using the 90% similarity criteria. This last set, which was used in most of the analyses reported, is referred to as the **90e **set. Table 1 summarizes the number of contigs and singletons obtained in each of those assemblies. The similarities between the three assemblies (**90**, **98 **and **90e) **are illustrated in Figure 1 a Venn diagram which shows that 72.68% of the raw sequencing reads were integrated into contigs common to all three assemblies, and 20.51% of the sequencing reads make up a shared pool of single sequencing reads (singletons). Therefore, differences between the assemblies can be explained by differential inclusion corresponding to 6.81% of the sequencing reads.

**Table 1:** Summary of sequence statistics for each assembly.

SET	Contigs	Singletons	TOTAL SEQs	GC%	LENGTHs [min/median/max/avg]			
**90**	52,885	137,213	190, 098	35.130	20	354	6812	355.78

**95**	52,501	137,077	189,578	---	---	---	---	---

**98**	52,321	137,353	189,674	35.127	20	354	6812	355.82

**90e**	53,867	138,766	192,633	35.108	20	358	7918	364.81

Set names are related to the corresponding homology level cutoff value (**90e **stands for 90% similarity including the set of NCBI Unigene ESTs). Contigs are the result of at least two sequencing reads, and singletons of only one read. GC content is the average value for all sequences. Sequence lengths are shown as minimum, median, maximum and average values in nucleotides for each set.

**Figure 2:**
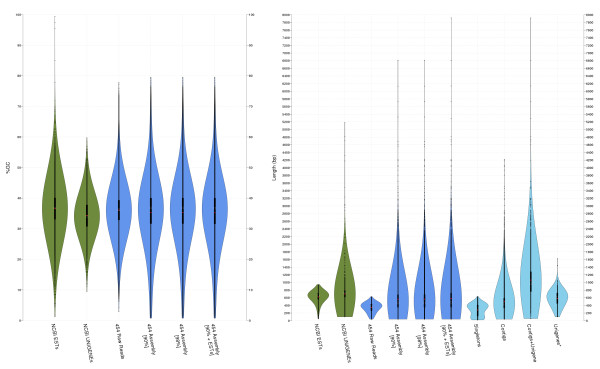
**GC content and length distributions of different assemblies**. Violin plots show the distribution of frequencies of a given variable in different datasets using a density kernel estimator [71]. White marks on the violin plots indicate the median value for a given variable, and the red points indicate the mean. The thick line marks the 25/75% inter-quartile range. GC content (left panel) distribution is quite similar in all the datasets, with higher frequencies around 35%. Nucleotide length (right panel) highlights the major differences between un-assembled (NCBI ESTs and the 454 raw reads) and assembled (NCBI UniGene, **90**, **98 **and **90e**) sets. The last four plots (light blue) show the length distribution for the component subsets of the **90e **assembly.

Average GC content and sequence length and their respective distributions were similar for all three assemblies (Table 1 and Figure 2). GC content is distributed around 35%, the expected value for coding sequences in this species. The **90e **length distribution shape was slightly shifted towards larger sequences. This shift was mainly due to a set of long sequences (> 800 bp) from the NCBI Unigene ESTs included in this assembly. This causal relationship was evident in the comparison of the following four subsets of sequences from the **90e **set (lightblue violin plots on Figure 2 right panel): singletons (136,271), contigs that do not contain UniGene ESTs (46,958), contigs including Unigene ESTs (6,909), and finally, Unigene ESTs not assembled into a contig (2,495).

### Mapping the 90e assembly onto the genome

**Figure 3:**
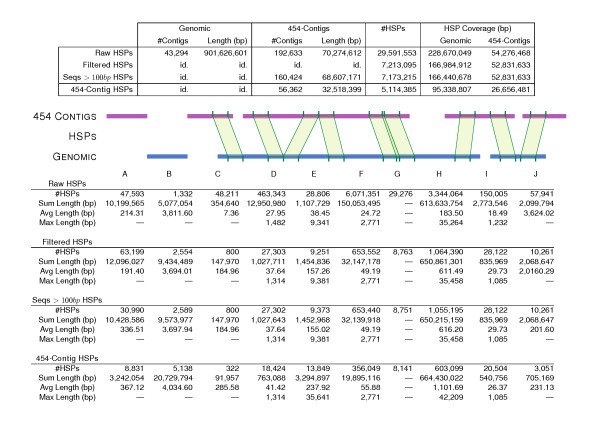
**Distribution of different HSP types from 90e over genome sequences**. The top table shows the total number of similarity hits, while the bottom table classifies the hits into different types of HSPs: A) 454 contigs not mapping to a genomic sequence; B) genomic contigs not mapping to a 454 contig; C and J) 454 contigs with an unmapped sequence on the left and right, respectively; D) missing sequence on 454 contigs corresponding to a putative gap in the assembly; E) contiguous HSPs on 454 contigs related to a genomic intron; F) co-linear unmapped sequences on both sequence sets; G) contiguous overlapping HSPs defining a larger similarity segment; H) unaligned genomic sequences between HSPs of two different 454 contigs, which can be interpreted as putative intergenic sequences; I) HSPs on 454 contigs supporting a pair of genomic contigs, which could then be merged into a larger genomic scaffold. All columns show HSP numbers--the '#HSPs' row--except for A and B, which correspond to number of sequences.

The **90e **assembly (192,633 sequences, 70,274,612 bases in total, average length of 365 bp per sequence) was aligned to scaffolds from the *S. mediterranea *WUSL genome assembly, version 3.1 [14] (43,294 sequences, 901,626,601 bases in total, average length of 20,8 kilobases per scaffold). Figure 3 shows all possible high-scoring segment pair (HSP) relationships between those two sequence sets. From almost 30 million initial HSPs, around 7 million were selected using a combination of thresholds, as described in the Methods section. Discarding singleton sequences in a second round of filtering further reduced the number of HSPs to 5 million, and HSP coverage dropped from 25.36% and 77.24%, for scaffolds and **90e **respectively, to 10.57% and 37.93%. However, when the total nucleotide length was considered only for the contigs (56,363 sequences, 32,518,399 bases in total, with an average of 577 bp per sequence), HSP coverage for **90e **rose to 81.97%. This means that most of the significant HSP hits are retained after the second round of filtering. In total, 8,831 contigs from **90e **did not map to the genomic contigs (3,242,054 bp that are completely novel and also transcribed, see column A in Figure 3). Conversely, 5,138 genomic contigs did not match a sequence from **90e **(column B). Of the **90e **contigs, 322 extended a genomic sequence from the left (column C) and 3,051 from the right (column J). The largest intergenic distance was 42,209 bp, with an average value of 1,102 bp (column H). The largest intron was estimated to be about 9,300 bp, the average length being 238 bp (column E). Finally, there were 20,504 HSPs connecting different genomic sequences via 8,604 different **90e **contigs (column I). Of the 8,831 **90e **contigs not found on the genome, 3,480 had a BLAST hit to the NCBI NR protein database (39.41%), and, of those, 2,401 had a hit to a protein with GO annotation (27.19%). After discarding abundant actin-like sequences (1,503), ATP/ADP transporter proteins (722) and sequences matching bacterial, protozoan or fungal genes (1,234), 71 **90e **contigs remained as new sequences not mapping on the genome (see Additional File 1).

**Figure 4:**
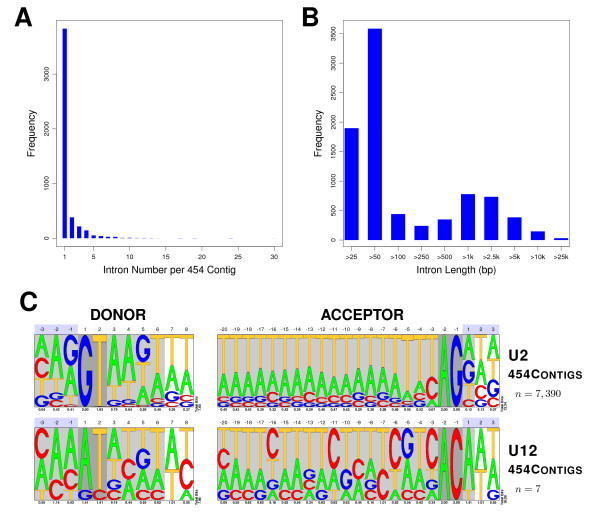
**Analysis of intronic features and splice sites on a set of 90e contigs**. A) Distribution of the number of putative introns per **90e **contig. B) Length distribution of putative introns. C) Pictograms summarizing the consensus donor and acceptor splice sites for the predicted introns. *n *corresponds to the number of intron sequences used to compute the nucleotide position weight matrices for the pictograms. Light grey shadowed regions correspond to the commonly used signal lengths for gene-finding, while dark grey ones define the nucleotide boundaries of the introns. Numbers below pictograms are the bit-scores that describe the information content per position.

In order to validate exonic structures, 6,226 **90e **contigs mapping 1-to-1 over genome sequences were selected. After re-aligning the **90e**/genomic sequence pairs, 4,739 contained at least one putative intron (see the corresponding splice sites boundaries in Additional File 2). In total 8,609 introns were retrieved from the genomic contigs. Figure 4 shows the number of introns per **90e **contig, as well as the length distribution for those introns. Pictograms summarize the nucleotide frequencies for the donor and acceptor splice sites, both for the U2 (canonical) and U12 (non-cannonical) introns. The splice sites patterns resemble those from other metazoan [19], taking into account that the genome of *S.mediterranea *is A/T-rich [16].

Also, 50 randomly picked **90e **contigs that either mapped or did not map to the genome were validated by RT-PCR (see Additional File 3 containing a list of the selected 90e contigs, as well as information on the primers used to amplify them). Additionally, 20 out of those 50 genes were further validated by sequencing. Finally, to further confirm the quality and coverage of the sequences from the **90e **dataset, the *S. mediterranea *genes already annotated in NCBI GenBank [20] were compared with those sequences. After discarding 18 S and 28 S ribosomal RNA genes and alpha-tubulins, 124 known genes were aligned to the **90e **sequences. In total, 108 of these genes had at least one significant similarity hit with one **90e **sequence, and two matched 5 sequences from **90e**. On average, the known genes had co-linear similarity hits against 1.32 different Smed454 sequences. Minimum and average similarities were 8.35% and 85.34% respectively, and 71 sequences had more than 95% similarity. Mean coverage dropped to 77.63% when each hit was considered separately. A summary of these similarity analyses is shown in Additional File 4.

### Browsing the Smed454 dataset

**Figure 5:**
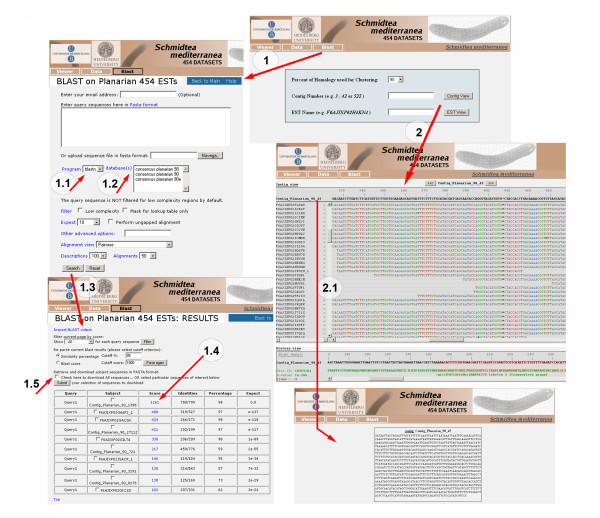
**The content of the Smed454 web site**. Screenshots of the pages that facilitate access to the three sequence assemblies (**90**, **98 **and **90e)**, including the page displaying alignments of raw reads. A BLAST interface, adapted from NBCI's toolkit, is also available for querying the sequences from the datasets. The web site is available at http://planarian.bio.ub.es/datasets/454/

In order to make the Smed454 dataset useful and accessible to the planarian and non-planarian communities, a public database is available via web [21]. The web site allows users to view contig assemblies along with their read alignments, and to perform BLAST searches against assembled sequences. The BLAST option in the home page menu (1 in Figure 5) allows the user to BLAST sequences of interest against the **90**, **98**, and **90e **databases (1.2 in Figure 5). Both nucleotide (BLASTN) and protein (BLASTP) searches can be performed (1.1 in Figure 5). Clicking on the Search button (1.3 in Figure 5) brings up a new window displaying a list of hits. When a score value is selected (1.4 in Figure 5), the alignment between the query sequence and the Smed454 hit is shown. The site also offers the option of downloading Smed454 sequences of interest (1.5 in Figure 5). The contig or singleton accession number can be browsed directly from the main home page (2 in Figure 5). When the user searches for a specific contig, a new window appears showing the alignment of all the sequencing reads assembled in that contig. At the bottom of that window, the result of a pre-computed BLAST on the contig consensus sequence is displayed. When a contig, singleton or read name is selected (2.1 in Figure 5), a new window will display the requested sequence. All raw and assembled sequence data are available from that web site too.

### Functional annotation of 90e sequences

**Figure 6:**
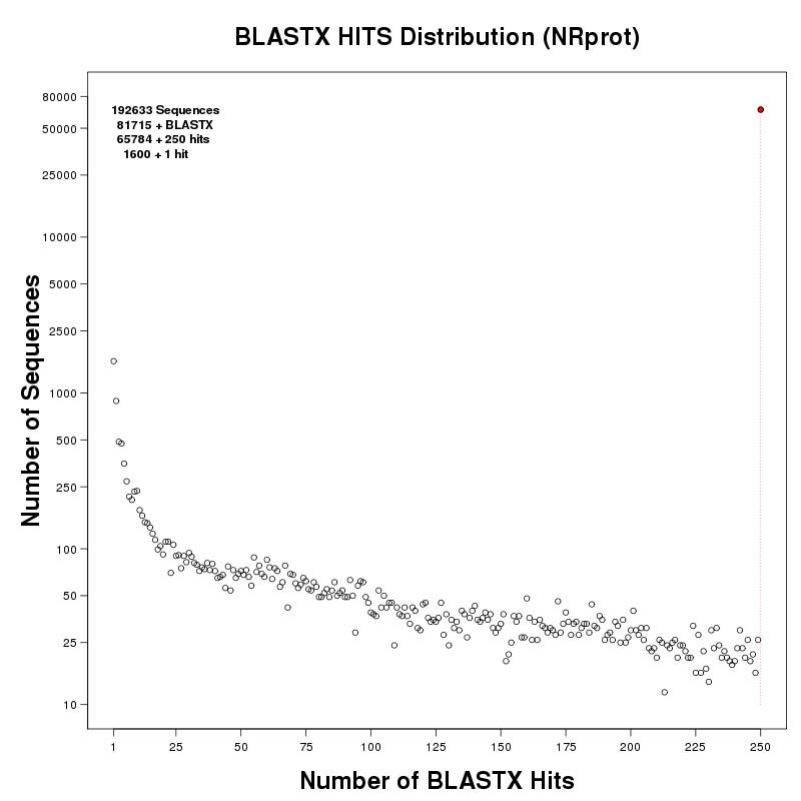
**Distribution of **BLASTX**hits of 90e sequences against NCBI NRprot**. Sequences from the **90e **dataset were compared against the NCBI NR protein database using BLASTX. The figure shows the distribution of the number of sequences binned by the number of HSPs they had. Y-axis in log scale.

In order to characterize the gene families that can be found on Smed454, we annotated the three datasets; we will focus on **90e **dataset here. In total, 42.42% of the sequences had a similarity hit with at least one protein sequence in the NCBI NR protein database [20]. Of these, almost two-thirds had 250 or more hits (see Figure 6), but the BLASTX output was limited to a maximum of 250 hits per **90e **sequence owing to the large number of HSPs reported by BLAST for some of them. The Gene Ontology (GO) [22] database was used to computationally annotate all the sequences (see Additional File 5 for **90**, **98**, and **90e **datasets) by mapping onto them the functional codes already assigned to known proteins from NCBI NR. Many of these sequence hits matched to a short ATP-binding domain, in most cases corresponding to proteins of the actins family. Consequently, that functional class, which was also anomalously over-represented, was discarded from the total number of annotations for the **90e **set, as shown in Table 2.

**Table 2:** Gene Ontology annotation for 90e set sequences.

**GO**	**Molecular Function**	**Count**	**Freq%**	**GO**	**Biological Process**	**Count**	**Freq%**	**GO**	**Cellular Component**	**Count**	**Freq%**
		
GO:0000166	nucleotide binding	54,823	---	---	---	---	---	GO:0043229	intracellular organelle	60,817	---
---	unannotated	9,709	---	---	unannotated	62,834	---	---	unannotated	11,131	---
GO:0016787	hydrolase activity	5,197	35.669	GO:0043170	macromolecule metabolic process	5,793	35.610	GO:0043234	protein complex	2,918	40.788
GO:0016740	transferase activity	2,030	13.933	GO:0022607	cellular component assembly	2,182	13.413	GO:0044424	intracellular part	2,314	32.346
GO:0043167	ion binding	1,323	9.080	GO:0006810	transport	1,213	7.456	GO:0031982	vesicle	819	11.448
GO:0003735	structural constituent of ribosome	874	5.999	GO:0006950	response to stress	1,070	6.577	GO:0044425	membrane part	469	6.556
GO:0005488	binding	761	5.223	GO:0050789	regulation of biological process	1,012	6.221	GO:0016020	membrane	210	2.935
GO:0016491	oxidoreductase activity	703	4.825	GO:0006807	nitrogen compound metabolic process	722	4.438	GO:0005622	intracellular	111	1.552
GO:0022857	transmembrane transporter activity	678	4.653	GO:0048869	cellular developmental process	655	4.026	GO:0044446	intracellular organelle part	91	1.272
GO:0030235	nitric-oxide synthase regulator activity	597	4.097	GO:0065009	regulation of molecular function	622	3.823	GO:0005576	extracellular region	63	0.881
GO:0043176	amine binding	580	3.981	GO:0009056	catabolic process	507	3.117	GO:0045211	postsynaptic membrane	21	0.294
GO:0005515	protein binding	532	3.651	GO:0044419	interspecies interaction between organisms	280	1.721	GO:0044420	extracellular matrix part	19	0.266
GO:0003676	nucleic acid binding	401	2.752	GO:0055114	oxidation reduction	236	1.451	GO:0043233	organelle lumen	18	0.252
GO:0005215	transporter activity	387	2.656	GO:0065008	regulation of biological quality	206	1.266	GO:0031012	extracellular matrix	16	0.224
GO:0016829	lyase activity	71	0.487	GO:0048856	anatomical structure development	193	1.186	GO:0042597	periplasmic space	15	0.210
GO:0016853	isomerase activity	55	0.377	GO:0051649	establishment of localization in cell	183	1.125	GO:0000267	cell fraction	15	0.210
GO:0048037	cofactor binding	52	0.357	GO:0044237	cellular metabolic process	182	1.119	GO:0044462	external encapsulating structure part	11	0.154
GO:0016874	ligase activity	49	0.336	GO:0023060	signal transmission	150	0.922	GO:0031975	envelope	8	0.112
GO:0004871	signal transducer activity	45	0.309	GO:0048870	cell motility	141	0.867	GO:0005615	extracellular space	7	0.098
GO:0003824	catalytic activity	32	0.220	GO:0008152	metabolic process	139	0.854	GO:0009986	cell surface	6	0.084
GO:0060589	nucleoside-triphosphatase regulator activity	32	0.220	GO:0023033	signaling pathway	107	0.658	GO:0043204	perikaryon	5	0.070
GO:0042277	peptide binding	28	0.192	GO:0044238	primary metabolic process	83	0.510	GO:0030427	site of polarized growth	4	0.056
GO:0022892	substrate-specific transporter activity	22	0.151	GO:0042221	response to chemical stimulus	69	0.424	GO:0042995	cell projection	3	0.042
GO:0019208	phosphatase regulator activity	12	0.082	GO:0006996	organelle organization	47	0.289	GO:0030312	external encapsulating structure	2	0.028
GO:0003712	transcription cofactor activity	12	0.082	GO:0007017	microtubule-based process	42	0.258	GO:0031594	neuromuscular junction	2	0.028
GO:0019207	kinase regulator activity	11	0.075	GO:0044281	small molecule metabolic process	39	0.240	GO:0045177	apical part of cell	2	0.028
GO:0008289	lipid binding	9	0.062	GO:0051301	cell division	37	0.227	GO:0019028	viral capsid	1	0.014
GO:0005201	extracellular matrix structural constituent	8	0.055	GO:0022613	ribonucleoprotein complex biogenesis	35	0.215	GO:0031252	cell leading edge	1	0.014
GO:0050840	extracellular matrix binding	6	0.041	GO:0019637	organophosphate metabolic process	34	0.209	GO:0044217	other organism part	1	0.014
GO:0061134	peptidase regulator activity	6	0.041	GO:0045184	establishment of protein localization	34	0.209	GO:0044297	cell body	1	0.014
GO:0030246	carbohydrate binding	6	0.041	GO:0009628	response to abiotic stimulus	23	0.141	GO:0044463	cell projection part	1	0.014
								
GO:0016248	channel inhibitor activity	5	0.034	GO:0019748	secondary metabolic process	21	0.129		**TOTAL**	7,154	
GO:0003702	RNA polymerase II transcription factor activity	5	0.034	GO:0009058	biosynthetic process	21	0.129				
GO:0005198	structural molecule activity	4	0.027	GO:0007155	cell adhesion	18	0.111				
GO:0016986	transcription initiation factor activity	4	0.027	GO:0061024	membrane organization	16	0.098				
GO:0042165	neurotransmitter binding	4	0.027	GO:0007275	multicellular organismal development	16	0.098				
GO:0003682	chromatin binding	3	0.021	GO:0016192	vesicle-mediated transport	12	0.074				
GO:0008430	selenium binding	3	0.021	GO:0043062	extracellular structure organization	10	0.061				
GO:0030234	enzyme regulator activity	2	0.014	GO:0034330	cell junction organization	9	0.055				
GO:0030528	transcription regulator activity	2	0.014	GO:0048609	reproductive process in a multicellular organism	9	0.055				
GO:0009055	electron carrier activity	2	0.014	GO:0007154	cell communication	8	0.049				
GO:0017056	structural constituent of nuclear pore	2	0.014	GO:0003008	system process	7	0.043				
GO:0017080	sodium channel regulator activity	2	0.014	GO:0016049	cell growth	7	0.043				
GO:0001871	pattern binding	2	0.014	GO:0016458	gene silencing	6	0.037				
GO:0019239	deaminase activity	2	0.014	GO:0008219	cell death	5	0.031				
GO:0043021	ribonucleoprotein binding	2	0.014	GO:0033036	macromolecule localization	4	0.025				
GO:0008538	proteasome activator activity	2	0.014	GO:0048610	reproductive cellular process	4	0.025				
GO:0030337	DNA polymerase processivity factor activity	1	0.007	GO:0051236	establishment of RNA localization	4	0.025				
GO:0031406	carboxylic acid binding	1	0.007	GO:0071684	organism emergence from protective structure	4	0.025				
GO:0042562	hormone binding	1	0.007	GO:0006955	immune response	4	0.025				
GO:0046906	tetrapyrrole binding	1	0.007	GO:0007049	cell cycle	4	0.025				
GO:0051540	metal cluster binding	1	0.007	GO:0009405	pathogenesis	4	0.025				
								
	**TOTAL**	14,570		GO:0009607	response to biotic stimulus	4	0.025				
				GO:0009791	post-embryonic development	4	0.025				
				GO:0048646	anatomical structure formation involved in morphogenesis	4	0.025				
				GO:0009790	embryonic development	3	0.018				
				GO:0015976	carbon utilization	2	0.012				
				GO:0032506	cytokinetic process	2	0.012				
				GO:0045103	intermediate filament-based process	2	0.012				
				GO:0070882	cellular cell wall organization or biogenesis	2	0.012				
				GO:0006413	translational initiation	2	0.012				
				GO:0009566	fertilization	2	0.012				
				GO:0001906	cell killing	1	0.006				
				GO:0043473	pigmentation	1	0.006				
				GO:0009987	cellular process	1	0.006				
				GO:0022402	cell cycle process	1	0.006				
				GO:0022411	cellular component disassembly	1	0.006				
				GO:0022415	viral reproductive process	1	0.006				
				GO:0023061	signal release	1	0.006				
				GO:0030030	cell projection organization	1	0.006				
				GO:0051606	detection of stimulus	1	0.006				
				GO:0000746	conjugation	1	0.006				
				GO:0007163	establishment or maintenance of cell polarity	1	0.006				
				GO:0009653	anatomical structure morphogenesis	1	0.006				
								
					**TOTAL**	16,268					

The most probable GO code in the three ontology categories--molecular function, biological process and cellular component-- for each sequence in the **90e **data set was selected. For simplicity, only level one and two codes are shown here in order.

Among the most abundant GO annotations at the biological process level, leaving aside metabolism-related features, 'response to stress' was found for 1,070 sequences (6.58%). This finding was expected because the original biological sample was a mixture of intact and regenerating planarians, both normal and irradiated. 'Regulation of biological process' was in the same range, with 1,012 sequences (6.22%). At the GO molecular function level, 'binding' was the most common annotation, although where possible a more specific annotion was provided by drilling down to the 2^nd ^level child annotations on the GO graph. It is interesting to find, among others, 3 'selenium binding' activities, since it has been reported that selenium may play an important role in cancer prevention, immune system function, male fertility, cardiovascular and muscle disorders, and prevention and control of the ageing process [23]. Finding selenium-binding proteins would be evidence of the presence of selenoproteins, which are thought to be responsible for most of the biomedical effects of selenium across eukaryota [24]. When looking at the cellular component level and discarding many of the 'intracellular organelles' due to their co-occurrence with 'nucleotide binding', there are a notably large number of 'protein complexes', 2,918 sequences (40.79%). With 819 sequences (11.45%), another important term on this level is 'vesicle', which correlates with secretory functions, apoptosis, and autophagy.

To prove the usefulness of the Smed454 dataset, we performed several searches on specific groups and gene families for which only scant data has been reported to date in planarians. Planarians are mainly known for their remarkable regenerative capabilities, which depend upon the presence of stem cells named neoblasts. Because of the unique properties of these cells, some studies have used a microarray-based strategy to detect neoblast-specific genes [25,26]. In our Smed454 dataset we were able to identify, in addition to known neoblast markers such as piwis, histones, bruli, vasa or tudor, several other genes annotated as involved in cell cycle or DNA damage and repair (Additional File 6). Within these gene set we find many cyclins and cell cycle division-related genes but also genes related to replication and chromosome maintenance. Finally, genes related to stress response and DNA damage were also identified, probably owing to the use of irradiated animals in the generation of the Smed454 dataset. In addition to these neoblast-related genes we were able to identify large collections of much less well-characterized families in planarians, such as neurotransmitter, peptide and hormone receptors, homeobox domain-containing genes, and genes related to eye function in other animals.

### Prediction of planarian transmembrane proteins

**Figure 7:**
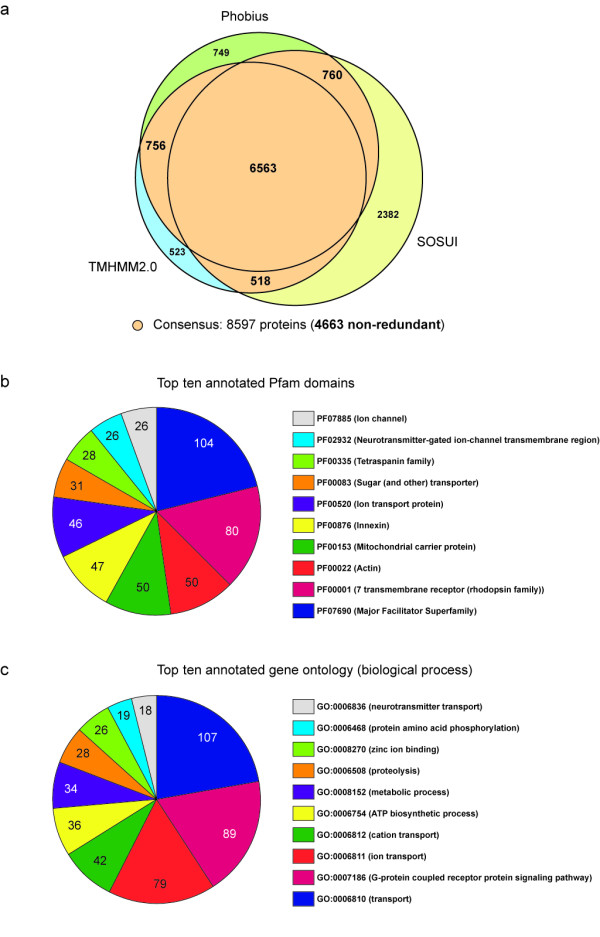
**Prediction of planarian transmembrane proteins and functional annotations**. A) Venn-diagram showing the overlap between predictions of transmembrane proteins generated by the Phobius, TMHMM2.0 and SOSUI programs for a set of 56,362 protein sequences translated from planarian ESTs. Only proteins predicted to contain one or more transmembrane domains by at least two programs (colored orange, 8,597 proteins, of which 4,663 are non-redundant) were considered for further analysis. B) Top ten PFAM domains and C) gene ontologies (biological process) for the 4,663 non-redundant transmembrane-proteins predicted. The figures indicate the number of proteins contained in a given annotation group.

Transmembrane (TM) proteins regulate a number of biological processes ranging from catalytic processes in intracellular and extracellular transport to cell-to-cell communication. TM proteins have become particularly interesting as many of them are key initiators of signal transduction pathways, and they can be easily manipulated by small molecule- or antibody-based drugs. To identify putative TM proteins from the planarian transcriptome, we mined the 454 dataset for putative TM protein-encoding messages (see Methods). Considering only the proteins that at least two application predicted would contain one or more transmembrane domains, resulted in a list of 8,597 predicted transmembrane proteins (see Figure 7a), which represents 15,3% of the complete protein database. Protein-BLAST searches were then used to align sequences to each other, and redundant sequences were removed from the predicted transmembrane set. The resulting database contained 4,663 sequences. Functional categorization using the UFO web-server [27] allowed us to assign PFAM protein families to 1,474 of the sequences and gene ontology classifications to 2,464. The top ten PFAM domains (~33% of all assignments) included, for example, the classifications for 'major facilitator superfamily' (a ubiquitous transporter family), '7 transmembrane receptor (rhodopsin family)' and 'ion transport protein' (see Figure 7b). The top ten gene ontology profiles (~49% of all assignments) included 'membrane' (cellular component), 'transport', and 'G-protein coupled receptor protein signalling pathway' (both biological processes, see Figure 7c). The enrichment of our database with proteins that have a predicted function in transport and receptor signalling supports the reliability of our approach. A complete list of the 4,663 predicted transmembrane proteins, the number of predicted transmembrane domains, predicted topology, and functional categorizations (PFAM and GO) are shown in Additional File 7.

### Neurotransmitter and hormone receptors in *Schmidtea mediterranea*

Despite our growing knowledge about how planarian neoblasts are regulated at the molecular level [9,25,26,28-31], we are still far from characterizing the complete repertoire of factors that control neoblast biology. Receptors for neurotransmitters, peptides and hormones are among the candidates for a role in the regulation of neoblast proliferation, differentiation and migration. In planarians, some of the data suggest that molecules such as dopamine [32,33], serotonin [34], substance P [35], somatostatin [36] and FMRFamide [37] can accelerate or delay the regeneration rate, probably by regulating neoblast proliferation and/or differentiation. A model has been proposed in which neoblasts express receptors for some of these factors, which in turn regulate the fate of these cells [35]. We found 288 contigs and singletons in the annotated Smed454 dataset with significant homology to neurotransmitter and hormone receptors (Table 3 and Additional File 8), providing a list of potentially interesting candidates.

**Table 3:** List of neurotransmitter, peptide and hormone receptor sequence candidates.

ID	BLASTX HIT	ACCESSION NUMBER	E-VALUE
90_1623	adiponectin receptor (Schistosoma mansoni)	XP_002577010.1	2,00E-103
90_11706	allatostatin receptor, putative (Ixodes scapularis)	XP_002414997.1	2,00E-18
90_9653	amine GPCR (Schistosoma mansoni)	XP_002576533.1	8,00E-25
P02IKPED	atrial natriuretic peptide receptor (Aedes aegypti)	XP_001652228.1	7,00E-28
P02HWID8	beta adrenergic receptor (Aedes aegypti)	XP_001651714.1	2,00E-18
90_17484	similar to bombesin-like peptide receptor (Ornithorhynchus anatinus)	XP_001514235.1	2,00E-12
90_19322	C1A receptor, putative (Ixodes scapularis)	XP_002405845.1	4,00E-04
90_4815	calcitonin receptor, isoform CRA_d (Rattus norvegicus)	AAA65964.1	3,00E-50
90_20672	cardioexcitatory receptor (Lymnaea stagnalis)	AAB92258.1	6,00E-11
90_6224	class b secretin-like g-protein coupled receptor GPRmth5 (Pediculus humanus)	XP_002427184.1	2,00E-10
P02IZJB4	similar to putative diuretic hormone receptor II (Nasonia vitripennis)	XP_001606711.1	4,00E-22
90_7506	type I dopamine receptor (Panulirus interruptus)	ABB87183.1	8,00E-69
90_6802	dopamine receptor type D2 (Apis mellifera)	NP_001011567.1	2,00E-27
90_8536	dro/myosuppressin receptor (Schistosoma mansoni)	XP_002570000.1	7,00E-26
90_9052	FMRFamide receptor (Culex quinquefasciatus)	XP_001849293.1	2,00E-17
P02GUXTP	similar to galanin receptor type I (Danio rerio)	XP_690480.1	3,00E-06
90_6830	glutamate receptor kainate (Schistosoma mansoni)	XP_002576035.1	3,00E-70
P02GLFYW	glutamate receptor NMDA (Schistosoma mansoni)	XP_002572261.1	3,00E-21
90_15092	glutamate receptor, ionotropic, AMPA 1b (Danio rerio)	NP_991293.1	5,00E-78
90_13524	metabotropic glutamate receptor (Schistosoma mansoni)	XP_002572726.1	1,00E-12
90_18656	gonadotropin-releasing hormone receptor type I (Capra hircus)	ABL76162.1	7,00E-04
90_4098	growth hormone secretagogue receptor (Schistosoma mansoni)	XP_002569813.1	7,00E-36
90_976	growth hormone-inducible transmembrane protein (Osmerus mordax)	AC008873.1	2,00E-51
90_6465	putative insulin receptor (Echinococcus multilocularis)	CAD30260.1	6,00E-61
90_7253	lung seven transmembrane receptor (Culex quinquefasciatus)	XP_001868443.1	1,00E-68
90_17047	metabotropic GABA-B receptor subtype, putative (Ixodes scapularis)	XP_002406087.1	4,00E-21
90_6512	natriuretic peptide receptor (Xenopus laevis)	NP_001083703.1	4,00E-158
90_12800	muscarinic acetylcholine (GAR) receptor (Schistosoma mansoni)	XP_002575679.1	2,00E-39
90_1507	Nicotinic acetylcholine receptor alpha 1 subunit (Aplysia californica)	AF467898_1	3,00E-44
90_223	neuroendocrine protein 7b2 (Schistosoma mansoni)	XP_002578500.1	6,00E-25
90_6302	similar to neuromedin U receptor 2 (Strongylocentrotus purpuratus)	XP_001200425.1	4,00E-27
90_29452	neuropeptide FF receptor 2 isoform 3 (Homo sapiens)	NP_001138228.1	2,00E-09
90_6772	neuropeptide F-like receptor (Schistosoma mansoni)	XP_002573542.1	1,00E-28
90_5995	neuropeptide Y receptor Y7 (Oncorhynchus mykiss)	ABB54774.1	9,00E-18
90_25975	octopamine receptor (Aplysia californica)	AAF37686.1	1,00E-25
90_8498	odorant receptor (Tetraodon nigroviridis)	CAG08888.1	6,00E-05
90_5999	similar to olfactory receptor 355 (Bos taurus)	XP_610381.4	1,00E-05
90_2541	P2Y purinergic receptor (Meleagris gallopavo)	AAA18784.1	2,00E-04
90_8537	P2X receptor subunit (Schistosoma mansoni)	XP_002580774.1	1,00E-72
90_19040	pituitary adenylate cyclase activating polypeptide receptor (Oncorhynchus mykiss)	NP_001118113.1	1,00E-08
90_28219	parathyroid hormone 2 receptor (Danio rerio)	AAI62580.1	3,00E-11
90_6836	peptide (allatostatin)-like receptor (Schistosoma mansoni)	XP_002572656.1	2,00E-66
90_7984	peptide (allatostatin/somatostatin)-like receptor (Schistosoma mansoni)	XP_002575539.1	2,00E-32
90_10769	progesterone receptor membrane component 1 (Danio rerio)	NP_001007393.1	7,00E-04
90_5450	progestin receptor membrane component 1 (Oryzias latines)	BAE47967.1	2,00E-28
P02GZGVI	prolactin releasing hormone receptor (Homo sapiens)	BAG36078.1	2,00E-06
P02I1U9K	pyrokinin-like receptor (Dermacentor variabilis)	ACC99623.1	2,00E-11
90_10680	Rhodopsin-like GPCR superfamily, domain-containing protein (Schistosoma japonicum)	CAX73015.1	6,00E-37
90_2955	rhodopsin-like orphan GPCR (Schistosoma mansoni)	XP_002579928.1	2,00E-42
90_27829	ryanodine receptor 44F (Schistosoma japonicum)	CAX69439.1	8,00E-16
90_14326	serotonin receptor-like planarian receptor 1 (Dugesia japonica)	BAA22404.1	3,00E-54
90_15981	serotonin receptor 7 (Dugesia japonica)	BAI44327.1	2,00E-14
90_11349	sex peptide receptor (Tribolium castaneum)	NP_001106940.1	5,00E-25
P02HBR62	SIFamide receptor (Apis mellifera)	NP_001106756.1	9,00E-10
90_19415	parathyroid hormone-related peptide receptor precursor (Tribolium castaneum)	XP_969953.1	7,00E-20
P02FKOY5	parathyroid hormone receptor 2, isoform CRA_c (Mus musculus)	EDL00229.1	3,00E-07
90_1140	somatostatin receptor (Culex quinquefasciatus)	XP_001859671.1	7,00E-43
P02JZNDR	tachykinin receptor 1 (Mus musculus)	NP_033339.2	2,00E-06
P02FL51R	thyroid hormone receptor (Schistosoma mansoni)	XP_002573733.1	2,00E-23
P02FHDMB	thyroid stimulating hormone receptor precursor (Canis lupus familiares)	NP_001003285.1	5,00E-04
90_3545	thyrotropin-releasing hormone receptor 1 (Catostomus commersonii)	AAG31763.1	2,00E-51
90_26294	tyramine receptor (Bombyx mori)	BAD11157.1	1,00E-11

### Homeobox-containing sequences in *Schmidtea mediterranea*

Since the first homeobox-containing genes were characterized in planarians [38], a large number of Hox and ParaHox genes that could be accommodated into the classical series of paralogous groups from Plhox1 to Plohox-9 and Xlox to cad/Cdx [39,40] have been described. Some of them show a differentially axial nested expression; while others are ubiquitously expressed [41-43]. Most of this work has been done in the planarians *Girardia tigrina *and *Dugesia japonica*. Recently, the first expression of an *S. mediterranea *Hox gene has been reported [44]. We identified 50 contigs and singletons with significant sequence similarity to homeobox gene sequences in the annotated Smed545 dataset (Table 4), including Hox genes and homeobox-containing genes, some already characterized in other planarian species.

**Table 4:** Complete list of homeobox-containing gene sequence candidates.

ID	BLASTX HIT	ACCESSION NUMBER	E-VALUE
F6AJIXP02J3PG4	arrowhead [Schistosoma mansoni]	XP_002575389	6,00E-09
90_9219	barh homeobox protein [Schistosoma mansoni]	XP_002571667	9,00E-26
F6AJIXP02J2YIH	brain-specific homeobox [Tribolium castaneum]	EFA05724	5,00E-05
90_23337	cut, isoform C [Drosophila melanogaster]	NP_001138174	3,00E-18
F6AJIXP02HF7ZO	cut, isoform C [Drosophila melanogaster]	NP_001138174	2,00E-10
90_8368	Cut-like homeobox 1 [Mus musculus]	AAH14289	2,00E-23
90_3019	distalless, Dlx-1 [Platynereis dumerilii]	CAJ38799	8,00E-07
90_14605	DjotxB [Dugesia japonica]	BAF80446	4,00E-65
F6AJIXP02FICZL	Eye absent protein [Dugesia japonica]	CAD89530	1,00E-74
F6AJIXP02IV6Y0	gsx family homeobox protein [Schistosoma mansoni]	XP_002574396	3,00E-12
90_24312	H6-like-homeobox [Drosophila melanogaster]	NP_732244	2,00E-15
90_8293	homeobox protein distal-less dlx [Schistosoma mansoni]	XP_002574393	4,00E-07
F6AJIXP02JJ1QK	Homeobox protein DTH-2 [Girardia tigrina]	Q00401	3,00E-40
90_8753	homeobox prox 1 [Danio rerio]	NP_956564	5,00E-19
90_12057	homeodomain protein Tlx [Capitella teleta]	ACH89436	1,00E-23
90_8083	Hox class homeodomain protein AbdBa Hox protein [Schmidtea mediterranea].	ABW79872	1,00E-26
90_7618	Hox class homeodomain protein DjAbd-Ba [Dugesia japonica]	BAB41079	2,00E-16
90_6369	Hox class homeodomain protein DjAbd-Bb [Dugesia japonica]	BAB41078	1,00E-108
F6AJIXP02ILMDY	Hox class homeodomain protein DjAbd-Bb [Dugesia japonica]	BAB41078	3,00E-33
F6AJIXP02HN15J_2	Hypothetical protein CBG18604 [Caenorhabditis briggsae]	XP_002638395	7,00E-05
90_28860	ladybird homeobox corepressor 1-like protein [Mus musculus	NP_001103213 XP_001479028	8,00E-33
90_6629	lim domain binding protein [Schistosoma mansoni]	XP_002576324	6,00E-05
F6AJIXP02GEYYP	lim domain homeobox 3/4 transcription factor [Saccoglossus kowalevskii)	NP_001158395	4,00E-23
90_10783	lim homeobox protein [Schistosoma mansoni]	XP_002579046	2,00E-13
90_11027	lim homeobox protein [Schistosoma mansoni]	XP_002579046	1,00E-26
90_10828	LIM homeobox transcription factor 1 alpha [Mus musculus]	EDL39177	2,00E-14
90_13775	LIM motif-containing protein kinase 1 [Schistosoma japonicum]	CAX72746	2,00E-11
90_9432	LIM-homeodomain protein AmphiLim1/5 [Branchiostoma floridae]	ABD59002	5,00E-05
90_8762	LIM-homeodomain transcription factor islet [Branchiostoma floridae	AAF34717	2,00E-15
90_6339	Nk1 protein [Platynereis dumerilii]	CAJ38797	1,00E-11
F6AJIXP02G077U	paired-like homeobox 2a [Danio rerio]	NP_996953	5,00E-16
90_6703	phtf [Drosophila melanogaster]	NP_610232	2,00E-55
90_25126	PLOX2-Dj [Dugesia japonica]	BAA77402	2,00E-42
90_21567	PLOX4-Dj [Dugesia japonica]	BAA77404	2,00E-21
90_23010	PLOX5-Dj [Dugesia japonica]	BAA77405	6,00E-22
F6AJIXP02IVOTI	PLOX5-Dj [Dugesia japonica]	BAA77405	1,00E-17
90_21710	pre-B-cell leukemia transcription factor 1 2 3 4 (pbx) [Schistosoma mansoni)	XP_002572195	2,00E-27
90_3405	PREDICTED: similar to UBX domain protein 4 [Hydra magnipapillata]	XP_002162754	2,00E-06
F6AJIXP02HI24E	PREP homeodomain-like protein [Schmidtea mediterranea]	ADB54565	2,00E-47
F6AJIXP02JSRJD	PREP homeodomain-like protein [Schmidtea mediterranea]	ADB54565	1,00E-32
F6AJIXP02GVFDM	prospero-like protein [Schistosoma mansoni]	XP_002578694	1,00E-21
F6AJIXP02IUJ5Q	prospero-like protein [Schistosoma mansoni]	XP_002578694	4,00E-25
F6AJIXP02HZIDG	short stature homeobox protein 2 isoform c [Homo sapiens	NP_001157150	5,00E-08
90_7545	SIX homeobox 2 [Gallus gallus]	NP_001038160	7,00E-36
F6AJIXP02HBGHT	SJCHGC06100 protein [Schistosoma japonicum]	AAW24487	6,00E-11
90_3395	UBX domain containing 8, isoform CRA_d [Mus musculus]	EDL41153	3,00E-14
90_1176	UBX domain-containing protein 4 [Mus musculus]	NP_080666	1,00E-06
90_2625	ubx6(yeast)-related [Schistosoma mansoni]	XP_002576054	2,00E-16
90_24438	visual system homeobox protein [Tribolium castaneum]	CAX64460	9,00E-23
F6AJIXP02G5JJX_1	Zn finger homeodomain 2 [Tribolium castaneum]	EFA01350	1,00E-05

### Eye genes in *Schmidtea mediterranea*

The structural simplicity of the planarian eye in conjunction with the regenerative abilities of these organisms provides a unique system for dissecting the genetic mechanisms that allow a simple visual structure to be built [45,46]. Despite great morphological differences, there is evidence that the early morphogenesis of animal eyes requires the regulatory activity of *Pax6*, *Sine oculis (Six)*, *Eyes absent (Eya) *and *Dachshund *(*Dach*), a gene network known as the retinal determination gene network (RDGN) [47-50]. Most of the genetic elements of the RDGN have been characterized in planarians [51-54]. In addition, the following planarian genes have been identified as being involved in eye regeneration: *Djeye53*, *Dj1020HH *[55]; *Smed-netR*, *Smed-netrin2 *[56]; *Gt/Smed/Dj ops *[46,57]; *Djsnap-25 *[58]; and *Smednos *[59]. In order to characterize new *S. mediterranea *eye network genes, we analyzed the Smed454 annotated dataset and found a collection of genes, ranging from transcription factors to eye-realizator genes, which have been implicated in eye development in other systems. These are good candidates for expanding our knowledge about the genetic network responsible for planarian eye regeneration (Table 5 and Additional File 9).

**Table 5:** List of eye-related gene sequence candidates.

ID	BLASTX HIT	**ACCESSION Nr**.	E-VALUE
90_7233	abl interactor 2 [Schistosoma japonicum]	CAX69750.1	6.00E-019
90_4001	adaptor-related protein complex [Schistosoma mansoni]	XP_002574891.1	3.00E-072
90_30923	arginine/serine-rich splicing factor [Schistosoma mansoni]	XP_002574990.1	2.00E-026
90_482	ATPase protein [Schistosoma japonicum]	AAW26203.1	3.00E-049
90_3152	beta-catenin-like protein 2 [Schmidtea mediterranea]	ABW79874.1	0
90_12909	BMP [Schmidtea mediterranea]	ABV04322.1	3.00E-090
90_120	cat eye syndrome protein [Schistosoma japonicum]	AAX27345.2	4.00E-035
P02FKNEB	CaTaLase family member (ctl-2) [Caenorhabditis elegans]	NP_001022473.1	1.00E-029
90_205	Chaperonin Containing TCP-1 family member (cct-3) [Caenorhabditis elegans]	NP_494218.2	1.00E-090
C90_6158	disks large homolog 1 isoform 1 [Homo sapiens]	NP_001091894.1	2.00E-027
P02GJNCV	extradenticle 1 protein [Schistosoma japonicum]	AAW24487.1	3.00E-013
P02JKJ4Z_2	eye53 [Dugesia japonica]	BAD20650.1	6.00E-016
90_8483	eyes absent protein [Dugesia japonica]	CAD89531.1	2.00E-064
90_651f	ascin protein [Schistosoma japonicum]	XP_002574990.1	5.00E-045
90_14368	heat shock protein 70 [Lumbricus terrestris]	ACB77918.1	4.00E-038
90_9533	Heparan sulfate 6-O-sulfotransferase 2 [Danio rerio]	AAH45453.1	1.00E-042
90_6564	histone-lysine n-methyltransferase suv9 [Schistosoma mansoni]	XP_002574171.1	3.00E-061
90_15456	homeodomain protein NK4 [Platynereis dumerilii]	ABQ10640.1	8.00E-023
90_12892	homeotic protein six3-alpha [Mus musculus]	S74256	1.00E-082
90_325	importin-7 [Culex quinquefasciatus]	XP_001843364.1	2.00E-147
90_4360	intraflagellar transport 57 homolog [Xenopus (Silurana) tropicalis]	NP_001016561.1	1.00E-044
90_11027	lim homeobox protein [Schistosoma mansoni]	XP_002579046.1	5.00E-027
90_8432	lozenge [Schistosoma mansoni]	XP_002580418.1	8.00E-032
90_8924	Male ABnormal family member (mab-21) [Caenorhabditis elegans]	NP_497940.2	1.00E-046
P02HSHWR	mothers against decapentaplegic homolog 4 [Mus musculus]	NP_032566.2	5.00E-018
90_5640	muscleblind-like protein [Schistosoma mansoni]	XP_002575346.1	3.00E-025
P02F0EF6	neurogenic differentiation [Platynereis dumerilii]	CAQ57533.1	2.00E-012
P02GMLJM	nuclear transcription factor X-box binding 1 (nfx1) [Schistosoma bovis]	XP_002577564.1	5.00E-014
90_828	phenylalanine hydroxylase [Caenorhabditis elegans]	AAD31643.1	2.00E-145
90_2925	protein [Schistosoma japonicum]	AAW24487.1	4.00E-126
90_7228	protein kinase [Schistosoma mansoni]	XP_002576342.1	2.00E-077
90_2256	Protein pob [Schistosoma japonicum]	CAX75988.1	4.00E-089
90_4436	Rab-protein 6 [Drosophila melanogaster]	NP_477172.1	8.00E-085
P02GENUT_1	retinaldehyde dehydrogenase 1 [Eleutherodactylus coqui]	ACE74542.1	8.00E-008
90_11988	runt protein [Branchiostoma lanceolatum]	AAN08565.1	4.00E-017
P02FN7BT	Septin-7 (CDC10 protein homolog) [Schistosoma japonicum]	CAX83064.1	3.00E-012
90_3747	serine/threonine protein kinase [Schistosoma mansoni]	XP_002580180.1	9.00E-094
P02FICZL	six1-2 protein [Dugesia japonica]	CAD89530.1	8.00E-86
P02IZDJZ_1	SRY-related HMG box B protein [Platynereis dumerilii]	CAY12631.1	3.00E-028
P02HE4J6	strabismus protein CBR-VANG-1 [Platynereis dumerilii]	CAJ26300.1	1.00E-006
90_9483	tetratricopeptide repeat protein 10 tpr10 [Schistosoma mansoni]	XP_002573898.1	4.00E-048
90_16088	tyrosine kinase [Schistosoma mansoni]	XP_002576978.1	2.00E-031
90_11388	ubiquitin conjugating enzyme E2 [Schistosoma mansoni]	XP_002578016.1	3.00E-053
90_1263	vacuolar ATP synthase proteolipid subunit 1 2 3 [Schistosoma japonicum]	XP_002571892.1	9.00E-049
90_12567	vermilion [Drosophila ananassae]	XP_001963597.1	2.00E-012
90_5500	white pigment protein [Drosophila melanogaster]	CAA26716.2	2.00E-020
90_13309	YY1 transcription factor [Schistosoma japonicum]	CAX73893.1	5.00E-049
90_10118	zinc finger protein 42 homolog [Homo sapiens]	NP_777560.2	6.00E-031
90_9460	14-3-3 zeta isoform [Schistosoma bovis]	AAT39382.1	2.00E-023
P02ILIK3	52-kD bracketing protein [Drosophila melanogaster]	CAA44483.1	1.00E-016

## Conclusions

The inherent complexity of the planarian genome and methodological difficulties initially prevented the complete genome assembly of *S. mediterranea*. High-throughput sequencing technologies are now well established and help molecular biologists to unravel the molecular components of organisms. We present a 454 sequencing dataset that can be used to decipher the transcriptome of the planarian *S. mediterranea*, an organism that has great potential for the study of regeneration processes.

We obtained more than half a million sequencing reads and assembled them into different datasets using a number of different similarity thresholds. The complete dataset has been made publicly available via web [21]. About 50,000 contigs in one of those sets (**90e) **were mapped against the most up-to-date genome scaffolds and to the set of known proteins from NCBI NR. Interestingly, we found a large number of transcribed sequences not covered by the genome sequence (more than 3 Mbp). The novel 454 contigs will allow us to extend current genomic sequences and connect up to 8,000 pairs of genome scaffolds. Furthermore, a preliminary analysis of the planarian splice sites was made on a collection of 454 contigs mapped univocally to the genome. Annotation of the sequences yielded a number of gene candidates in different functional categories that will be useful for further experimental studies. However, many of the novel contigs have no similarity to known proteins and will require further validation if we want to understand the transcriptional inventory of the planarian at a functional level. We also provided a preliminary gene annotation for *S. mediterranea*, focusing our rankings on four different gene families; these serve as applied examples of the usefulness of this new sequence resource.

## Methods

### Animals and RNA isolation

*Schmidtea mediterranea *from the BCN-10 clonal line were used. Animals were starved one week prior to experiments and irradiated at a lethal dose of 100Gy. Total RNA was isolated from a mixed sample of planarians that contained non-irradiated intact and regenerating planarians (1, 3, 5 and 7 days of regeneration) as well as irradiated intact and regenerating animals (1, 3, 5 and 7 days of regeneration). RNA was extracted with TRIzol^® ^(Invitrogen) following the manufacturer's instructions.

### cDNA library construction and 454 sequencing

First, 5 μg of total RNA was used to construct a cDNA library. RNA quality was assessed in a Bioanalyzer 2100 (Agilent-Bonsai Technologies). 5 μg of full-length double-stranded cDNA was then processed by the standard Genome Sequencer library-preparation method using the 454 DNA Library Preparation Kit (Titanium chemistry) to generate single-stranded DNA ready for emulsion PCR (emPCR™). The cDNA library was then nebulized according to the fragmentation process used in the standard Genome Sequencer shotgun library preparation procedure. The cDNA library was sequenced according to GS FLX technology (454/Roche). Reads were assembled by MIRA[60] version 3 using enhanced 454 parameters.

### Mapping to genomic and functional annotation

BLAT[61] was used with default parameters to map the Smed454 **90e **dataset on the *S. mediterranea *draft genome assembly v3.1 [14] since the 454 sequences should be very similar to the corresponding genomic sequences, except for the lack of introns. Perl scripts were developed to classify all HSPs into the categories shown in Figure 3. **90e **contigs having two or more collinear HSPs covering more than 100bp of the contig, and for which HSPs had more than 90% identity to the genomic contigs and length of the HSP larger than 50 bp, were chosen as 1-to-1 matches to genome. Once the sequences of the 90e/genomic contig pairs were retrieved, exonerate[62] was used to refine the alignments over the splice sites (using as parameters model = est2genome and bestn = 10). Perl scripts were used to retrieve the splice sites coordinates from exonerate output, as well as the sequences from genomic contigs. After clipping the donor and acceptor splice sites for each intron, nucleotide frequencies were computed and the corresponding position weight matrices for U2/U12 sites were drawn as pictograms using compi[19]. Known *S. mediterranea *genes were compared with contigs from **90e **using BLASTN[63] with the following cut-offs: e-value = 0.001, identity score > 80%, HSP length > 50 bp.

GO functional annotation was computed on the BLASTX[63] results of the three assembly datasets (**90**, **98**, and **90e) **against all proteins from NCBI NR. BLASTX parameters were set to e-value = 10e-25 and maximum number of descriptions and alignments to report = 250, which produced around 26 million HSPs for each set. After that, only HSPs with a minimum length of 80 bp and a similarity score of at least 80% were considered. GO annotation was performed on those HSPs using the e-value selection criteria and supporting sequences described for Blast2GO[64]. Further Perl scripts were used to summarize the data shown in Table 2 and Additional File 3.

### RT-PCR

In order to validate the expression of a random subset of novel 454 transcripts, RT-PCRs were performed on planarian cDNA generated with Superscript III (Invitrogen) following the manufacturer's instructions. Additional File 3 includes a list of the contigs validated and the primers used for each of them.

### Prediction of transmembrane proteins from ESTs

A total of 53,867 assembled ESTs (**90e **database) and 2,495 additional mRNAs were translated into all six reading frames using the 'transeq' program from the EMBOSS package [65]. The longest open reading frame for each EST/mRNA was then extracted and used as a protein database (containing 56,362 protein sequences overall) for the prediction of membrane-spanning proteins. We followed an approach described by Almen et al. [66] basing our analysis on consensus predictions of alpha-helices and using three applications: Phobius[67], TMHMM2.0[68], and SOSUI[69]. Phobius and TMHMM2.0 both use hidden Markov models based on different training sets to predict membrane topology. SOSUI evaluates proteins for their hydrophobic and amphiphilic properties to make its predictions. The use of all three programs should improve prediction accuracy. We first ran Phobius, which can predict both transmembrane helices and signal peptides. Signal peptide sequences are similar to transmembrane segments owing to their hydrophobic nature [70]. To avoid false positive predictions, we excluded signal peptides before running TMHMM2.0 and SOSUI.

## Abbreviations

bp: base pairs (nucleotide length unit); EST: Expressed Sequence Tag; GC%: percent of guanine+cytosine sequence content; HSP: High-scoring Segment Pair; GO: Gene Ontology; WUSL: Washington University in St Louis; TM: transmembrane; RDGN: retinal determination gene network; Gy: gray.

## Authors' contributions

JFA performed the computational analyses on the assemblies, the GO characterization, the mapping into the genome and the analysis of spliced sites, and prepared all the corresponding figures and tables. GRE analyzed the coverage of known annotated genes and generated the corresponding table. TH performed the sequence analysis of planarian transmembrane proteins, generated the corresponding figure and table and designed primers for RT-PCRs. GRE, SF, FC and KB designed the primers and performed the RT-PCRs. FC and SF analyzed the annotated data to characterize the neurotransmitter, peptide and hormone receptors and prepared the corresponding tables. ES and BC analyzed the annotated data to characterize homeobox-containing and eye-related genes and prepared the corresponding tables. JFA, KB, FC and ES conceived of the study, participated in its design and coordination, and helped draft the manuscript. All authors read and approved the final manuscript.

## Supplementary Material

Additional file 1 **GO annotation for 90e contigs not mapping onto the WUSL 3.1 genome assembly**. 8,831 **90e **contigs were not found in the genome. 3,480 had a BLASTX hit to a sequence of NCBI NRprot; yet only 2,401 had a hit to a protein functionally annotated in the GO database. This file contains the description of the best HSP for 71 of those annotated contigs, after filtering out as described above. (Header: CONTIG ID = Smed454 sequence identifier, E-VALUE = BLASTX HSP E-value, ALN_SCORE = HSP alignment score, IDENTITIES = number of identical amino acids, POSITIVES = number of similar amino acids, SEQUENCE ID = Protein sequence identifier, ACCESSION NUMBER = Protein sequence full accession number, SEQUENCE DESCRIPTION = Full protein GenBank description). Click here for file

Additional file 2 **Splice sites for a subset of Smed454 sequences mapped onto the *Schmidtea mediterranea *genome**. (Header: GID = Genomic contig IDentifier from WUSLv3.1 genome assembly--including the start and end nucleotide coordinates for the complete match--, CIG=**90e **contig IDentifier, INTNUM = Intron number within the **90e **contig, EXO = splice signals found by exonerate, ORI = sequence orientation--here -1 means that the match was found on the reverse strand of the genomic contig--, CEXO = corrected splice site signals after reverse complementing the genomic sequence when required, ILEN = Intron length in bp, IORI = Intron start--relative to the match coordinates--, IEND = Intron end--relative to the match coordinates--, STRAND, SSSEQ = Splice sites sequences--where a point separates three nucleotides from the 5' and 3' exons, and the three dots in the middle denote intron sequence not shown for clarity--). Click here for file

Additional file 3 **List of 90e transcripts validated by RT-PCR**. (Header: # = Number, CONTIG=**90e **contig ID, PRIMER_FORWARD = 5' to 3' sequence of the forward primer used, REVERSE_FORWARD = 5' to 3' sequence of the reverse primer used, AMPLICON SIZE = Size amplified in bp, SET = refers to the subset of origin of the **90e **contig: no hit genome, hit genome, - blast (no BLASTX hit), +blast (BLASTX hit)). Click here for file

Additional file 4 **Smed454 sequences matching known *Schmidtea mediterranea *genes**. (Header: ACCESSION NUMBER = Known gene sequence identifier as target, NAME = Description for that sequence, LENGTH = Nucleotide length for that sequence, A&T CONTENT = Sequence composition, 454 **90e **CONTIG/SINGLETON = Smed454 sequence identifier as query, LENGTH = Nucleotide sequence length for this sequence, ALIGNMENT LENGTH = HSP length, START = Start nucleotide of alignment on target, END = Final nucleotide of alignment on target, IDENTITY = Identity score, BITSCORE = Alignment bit score, E-VALUE = HSP BLAST e-value, HIT LENGTH = Un-gapped length of the alignment on the target, %COVERAGE = Sum of co-linear HSPs on target coordinates divided by the total length of the target, #SEQs = Number of co-linear HSPs considered, avg%COV = The coverage divided by the number of co-linear HSPs). Click here for file

Additional file 5 **Gene Ontology for all three Smed454 sets: 90, 98 and 90e**. Level one and two GO codes are shown in order to simplify the listings. Although there are small changes in GO frequencies, annotation is consistent throughout all three sets. (Header: GO = Gene Ontology unique identifier, Count = Number of sequences with a given GO annotation, Freq% = Frequencies for every GO annotation. The total shown does not include the un-annotated and over-represented features, that is, the first two rows on each table). Click here for file

Additional file 6 **List of cell cycle, cell division, DNA repair or DNA damage candidates**. Short list of candidates annotated as genes involved in cell cycle, cell division, DNA repair or DNA damage. (Header: ID = Smed454 sequence identifier, BLASTX HIT = Description of the best sequence hit, ACCESSION NUMBER = Sequence identifier of the best sequence hit, E-VALUE = BLASTX e-value for that sequence hit). Click here for file

Additional file 7 **Summary report for the consensus set of 4,663 predicted transmembrane proteins including functional annotations**. (Header: Sequence_ID = Protein sequence identifier, Sequence_AA = Amino acid sequence, Length[aa] = Length of amino acid sequence, Phobius_TM = Phobius prediction of number of transmembrane domains, Phobius_SP = Phobius prediction of signal peptide, Phobius_Top = Phobius prediction of membrane topology, TMHMM_TM = TMHMM2.0 prediction of number of transmembrane domains, TMHMM_Top = TMHMMv2.0 prediction of membrane topology, SOSUI_TM = SOSUI prediction of number of transmembrane domains, SOSUI_Top = SOSUI prediction of membrane topology, UFO_PFAM = UFO annotation of Pfam protein families, UFO_GO = UFO annotation of gene ontologies). Click here for file

Additional file 8 **List of neurotransmitter, peptide and hormone receptor sequence candidates**. Complete complement of Smed454 dataset contigs and singletons showing homology to neurotransmitter and hormone receptors, totalling 287 sequences. (Header: ID = Smed454 sequence identifier, BLASTX HIT = Description of the best sequence hit, ACCESSION NUMBER = Sequence identifier of the best sequence hit, E-VALUE = BLASTX e-value for that sequence hit). Click here for file

Additional file 9 **List of eye-related gene sequence candidates**. Complete complement of Smed454 dataset contigs and singletons showing homology to eye-related genes, totalling 95 sequences. (Header: ID = Smed454 sequence identifier, BLASTX HIT = Description of the best sequence hit, ACCESSION NUMBER = Sequence identifier of the best sequence hit, E-VALUE = BLASTX e-value for that sequence hit). Click here for file
