# Supplementary material for: Smed454 dataset: unravelling the transcriptome of Schmidtea mediterranea
Source: BMC Genomics. 2010 Dec 31;11:731. doi: 10.1186/1471-2164-11-731 (PMC3022928; doi:10.1186/1471-2164-11-731)
Supplement: Additional file 9 — List of eye-related gene sequence candidates. Complete complement of Smed454 dataset contigs and singletons showing homology to eye-related genes, totalling 95 sequences. (Header: ID = Smed454 sequence identifier, BLASTX HIT = Description of the best sequence hit, ACCESSION NUMBER = Sequence identifier of the best sequence hit, E-VALUE = BLASTX e-value for that sequence hit). [file 1471-2164-11-731-S9.DOC]

| **Selected** **list of eye related genes from Smed 454 database annotated by GO** | | | |
| --- | --- | --- | --- |
| **ID** | **BLASTX HIT** | **ACCESSION NUMBER** | **E-VALUE** |
| 90_7233 | abl interactor 2 [Schistosoma japonicum] | CAX69750.1 | 6.00E-019 |
| 90_4001 | adaptor-related protein complex [Schistosoma mansoni] | XP_002574891.1 | 3.00E-072 |
| 90_1422 | adaptor-related protein complex [Schistosoma mansoni] | XP_002574891.1 | 3.00E-065 |
| 90_3607 | adenylyl cyclase-associated protein [Aedes aegypti] | XP_001663284.1 | 3.00E-103 |
| 90_1728 | Adenylyl cyclase-associated protein 1 [Schistosoma japonicum] | CAX69899.1 | 2.00E-073 |
| P02H134 | Cankyrin 23/unc44 [Schistosoma mansoni] | XP_002574584.1 | 7.00E-011 |
| P02F1FXW | complex subunit delta-1 isoform 2 [Homo sapiens] | NP_003929.4 | 1.00E-031 |
| 90_5588 | ap3d1 protein [Xenopus (Silurana) tropicalis] | AAH80909.1 | 8.00E-141 |
| 90_30923 | arginine/serine-rich splicing factor [Schistosoma mansoni] | XP_002574990.1 | 2.00E-026 |
| 90_2733 | arginine/serine-rich splicing factor [Schistosoma mansoni] | XP_002574990.1 | 1.00E-019 |
| 90_7324 | argininosuccinate lyase [Xenopus (Silurana) tropicalis] | NP_001106586.1 | 4.00E-036 |
| 90_33357 | Atp6v0c protein [Mus musculus] | AAH50939.1 | 1.00E-011 |
| 90_482 | ATPase protein [Schistosoma japonicum] | AAW26203.1 | 3.00E-049 |
| 90_3152 | beta-catenin-like protein 2 [Schmidtea mediterranea] | ABW79874.1 | 0 |
| 90_12909 | BMP [Schmidtea mediterranea] | ABV04322.1 | 3.00E-090 |
| 90_120 | cat eye syndrome protein [Schistosoma japonicum] | AAX27345.2 | 4.00E-035 |
| P02FKNEB | CaTaLase family member (ctl-2) [Caenorhabditis elegans] | NP_001022473.1 | 1.00E-029 |
| 90_205 | Chaperonin Containing TCP-1 family member (cct-3) [Caenorhabditis | NP_494218.2 | 1.00E-090 |
| P02JUWEX | Chaperonin Containing TCP-1 family member (cct-3) [Caenorhabditis briggsae] | NP_494218.2 | 5.00E-009 |
| 90_10138 | dead eye [Oncorhynchus mykiss] | ABA42832.1 | 2.00E-010 |
| C90_6158 | disks large homolog 1 isoform 1 [Homo sapiens] | NP_001091894.1 | 2.00E-027 |
| P02GJNCV | extradenticle 1 protein [Schistosoma japonicum] | AAW24487.1 | 3.00E-013 |
| P02JKJ4Z_2 | eye53 [Dugesia japonica] | BAD20650.1 | 6.00E-016 |
| 90_16994 | eyes absent protein [Dugesia japonica] | CAD89531.1 | 1.00E-032 |
| 90_8483 | eyes absent protein [Dugesia japonica] | CAD89531.1 | 2.00E-064 |
| 90_493 | fascin protein [Schistosoma japonicum] | AAX24244.2 | 5.00E-023 |
| 90_651 | fascin protein [Schistosoma japonicum] | XP_002574990.1 | 5.00E-045 |
| P02FTUYA | heat shock cognate 70-kd protein [Danio rerio] | NP_571472.1 | 2.00E-032 |
| 90_14368 | heat shock protein 70 [Lumbricus terrestris] | ACB77918.1 | 4.00E-038 |
| 90_9533 | Heparan sulfate 6-O-sulfotransferase 2 [Danio rerio] | AAH45453.1 | 1.00E-042 |
| 90_6564 | histone-lysine n-methyltransferase suv9 [Schistosoma mansoni] | XP_002574171.1 | 3.00E-061 |
| 90_15456 | homeodomain protein NK4 [Platynereis dumerilii] | ABQ10640.1 | 8.00E-023 |
| 90_12892 | homeotic protein six3-alpha [Mus musculus] | S74256 | 1.00E-082 |
| 90_325 | importin-7 [Culex quinquefasciatus] | XP_001843364.1 | 2.00E-147 |
| 90_4360 | intraflagellar transport 57 homolog [Xenopus (Silurana) tropicalis] | NP_001016561.1 | 1.00E-044 |
| P02H6ZXE | Jab1/MPN domain metalloenzyme (M67 family) [Schistosoma mansoni] | XP_002573204.1 | 6.00E-028 |
| 90_7610 | Kruppel-like zinc finger protein putative [Ixodes scapularis] | XP_002435294.1 | 0 |
| 90_11027 | lim homeobox protein [Schistosoma mansoni] | XP_002579046.1 | 5.00E-027 |
| 90_8762 | lim homeobox protein [Schistosoma mansoni] | XP_002579046.1 | 1.00E-022 |
| 90_5835 | low-Mr GTP-binding protein Rab32 [Homo sapiens] | AAB02833.1 | 5.00E-057 |
| P02FGTKN | lozenge [Schistosoma mansoni] | XP_002580418.1 | 1.00E-005 |
| 90_6286 | lozenge [Schistosoma mansoni] | XP_002580418.1 | 1.00E-025 |
| 90_8432 | lozenge [Schistosoma mansoni] | XP_002580418.1 | 8.00E-032 |
| 90_9238 | MAD homolog 4 [Schistosoma japonicum] | CAX73585.1 | 7.00E-105 |
| 90_8924 | Male ABnormal family member (mab-21) [Caenorhabditis elegans] | NP_497940.2 | 1.00E-046 |
| 90_11109 | merlin/moesin/ezrin/radixin [Culex quinquefasciatus] | XP_001849773.1 | 5.00E-012 |
| P02IK0US | mindbomb 1 [Drosophila melanogaster] | NP_648826.2 | 2.00E-007 |
| P02JGCCN_1 | mindbomb homolog 1 [Xenopus (Silurana) tropicalis] | NP_001123407.1 | 3.00E-013 |
| P02HSHWR | mothers against decapentaplegic homolog 4 [Mus musculus] | NP_032566.2 | 5.00E-018 |
| 90_13047 | muscleblind-like protein [Schistosoma mansoni] | XP_002575346.1 | 4.00E-006 |
| 90_9732 | muscleblind-like protein [Schistosoma mansoni] | XP_002571979.1 | 6.00E-022 |
| 90_5640 | muscleblind-like protein [Schistosoma mansoni] | XP_002575346.1 | 3.00E-025 |
| 90_15752 | myosin heavy chain [Lethenteron japonicum] | BAD01608.1 | 2.00E-013 |
| P02HVS1P | Pneurobeachin [Culex quinquefasciatus] | XP_001848700.1 | 2.00E-014 |
| P02F0EF6 | neurogenic differentiation [Platynereis dumerilii] | CAQ57533.1 | 2.00E-012 |
| 90_9903 | neuronal cyclin-dependent kinase 5 [Schistosoma japonicum] | CAX70385.1 | 1.00E-077 |
| P02GMLJM | nuclear transcription factor X-box binding 1 (nfx1) [Schistosoma bovis] | XP_002577564.1 | 5.00E-014 |
| 90_4698 | pelota [Schistosoma mansoni] | XP_002571764.1 | 4.00E-100 |
| P02HHE5A | pelota [Schistosoma mansoni] | XP_002571764.1 | 3.00E-010 |
| 90_6093 | pelota homolog (Drosophila) [Xenopus tropicalis] | CAJ83117.1 | 1.00E-014 |
| 90_828 | phenylalanine hydroxylase [Caenorhabditis elegans] | AAD31643.1 | 2.00E-145 |
| 90_5961 | phosphatase Slingshot [Culex quinquefasciatus] | XP_001869860.1 | 3.00E-065 |
| 90_5826 | pnt [Drosophila sechellia] | XP_002032331.1 | 4.00E-035 |
| 90_2925 | protein [Schistosoma japonicum] | AAW24487.1 | 4.00E-126 |
| 90_4163 | protein CLN8 [Homo sapiens] | NP_061764.2 | 6.00E-037 |
| 90_7228 | protein kinase [Schistosoma mansoni] | XP_002576342.1 | 2.00E-077 |
| 90_2256 | Protein pob [Schistosoma japonicum] | CAX75988.1 | 4.00E-089 |
| 90_4436 | Rab-protein 6 [Drosophila melanogaster] | NP_477172.1 | 8.00E-085 |
| 90_799 | Ras GTPase-activating protein-binding protein 1 [Schistosoma japonicum] | CAX73784.1 | 5.00E-026 |
| P02GENUT_1 | retinaldehyde dehydrogenase 1 [Eleutherodactylus coqui] | ACE74542.1 | 8.00E-008 |
| 90_11988 | runt protein [Branchiostoma lanceolatum] | AAN08565.1 | 4.00E-017 |
| P02JAE26 | septin [Schistosoma mansoni] | XP_002577304.1 | 4.00E-012 |
| P02FN7BT | Septin-7 (CDC10 protein homolog) [Schistosoma japonicum] | CAX83064.1 | 3.00E-012 |
| P02HFHDH | serine/threonine protein kinase [Schistosoma mansoni] | XP_002573741.1 | 2.00E-022 |
| 90_8596 | serine/threonine protein kinase [Schistosoma mansoni] | XP_002573741.1 | 9.00E-078 |
| 90_13380 | serine/threonine protein kinase [Schistosoma mansoni] | XP_002580180.1 | 1.00E-030 |
| 90_3747 | serine/threonine protein kinase [Schistosoma mansoni] | XP_002580180.1 | 9.00E-094 |
| P02FICZL | six1-2 protein [Dugesia japonica] | CAD89530.1 | 8.00E-86 |
| P02IZDJZ_1S | SRY-related HMG box B protein [Platynereis dumerilii] | CAY12631.1 | 3.00E-028 |
| P02HE4J6 | strabismus protein CBR-VANG-1 [Platynereis dumerilii] | CAJ26300.1 | 1.00E-006 |
| 90_9483 | tetratricopeptide repeat protein 10 tpr10 [Schistosoma mansoni] | XP_002573898.1 | 4.00E-048 |
| 90_16088 | tyrosine kinase [Schistosoma mansoni] | XP_002576978.1 | 2.00E-031 |
| 90_11388 | ubiquitin conjugating enzyme E2 [Schistosoma mansoni] | XP_002578016.1 | 3.00E-053 |
| 90_19356 | ubiquitin--protein ligase edd [Schistosoma mansoni] | XP_002576566.1 | 6.00E-026 |
| 90_3746 | ubiquitin-conjugating enzyme morgue [Schistosoma mansoni] | XP_002579371.1 | 3.00E-039 |
| 90_1263 | vacuolar ATP synthase proteolipid subunit 1 2 3 [Schistosoma japonicum] | XP_002571892.1 | 9.00E-049 |
| 90_10234 | vacuolar ATP synthase subunit F [Schistosoma mansoni] | XP_002573751.1 | 1.00E-042 |
| P02FGZ0N | Vacuolar protein sorting-associated protein 28 homolog [Schistosoma bovis] | CAX70158.1 | 7.00E-010 |
| 90_12567 | vermilion [Drosophila ananassae] | XP_001963597.1 | 2.00E-012 |
| 90_5500 | white pigment protein [Drosophila melanogaster] | CAA26716.2 | 2.00E-020 |
| 90_4830 | Winged helix transcription factor XFD-11 [Xenopus laevis] | AAI69733.1 | 2.00E-045 |
| 90_13309 | YY1 transcription factor [Schistosoma japonicum] | CAX73893.1 | 5.00E-049 |
| 90_10118 | zinc finger protein 42 homolog [Homo sapiens] | NP_777560.2 | 6.00E-031 |
| 90_9460 | 14-3-3 zeta isoform [Schistosoma bovis] | AAT39382.1 | 2.00E-023 |
| P02ILIK3 | 52-kD bracketing protein [Drosophila melanogaster] | CAA44483.1 | 1.00E-016 |
